# A protocol for lumbar spine surgery under spinal anesthesia in resource limited countries: illustrative case series

**DOI:** 10.1097/MS9.0000000000002824

**Published:** 2025-01-09

**Authors:** Sulaiman Jemal Muzien

**Affiliations:** Anesthesia Department, Addis Ababa University, Collage of Health Science, Addis Ababa, Ethiopia

**Keywords:** case series and neurosurgery, lumbar spine, protocol, spinal anesthesia, spinal surgery

## Abstract

**Introduction:**

Lumbar spine surgery can be performed under either general or spinal anesthesia. Numerous studies highlight the benefits of spinal anesthesia (SA), including cost-effectiveness, reduced anesthesia-related complications, and suitability for patients who do not favor general anesthesia (GA). Those informal case series emphasizes the advantages of SA and introduces a working protocol tailored for resource-limited countries (RLCs).

**Presentation of case:**

Two patients, aged 35 and 58, underwent spinal surgery using SA combined with local infiltration for the skin and facet joints. We implemented a new protocol believed to be beneficial in RLCs. In both cases, intraoperative vital signs remained stable, and there was effective pain control postoperatively.

**Clinical discussion:**

Spine surgery under SA has gained acceptance due to evidence indicating reduced perioperative risks and lower opioid consumption, alongside decreased healthcare costs. Although there are suggested protocols for SA in spine surgery, many are not applicable in RLCs. Our case series demonstrate similar advantages, suggesting that the protocol used in this study may be helpful. Despite its benefits, SA for spine surgery still faces resistance and has not been widely adopted in many neurosurgery centers.

**Conclusion:**

The study aim to outline essential steps for initiating SA for spine surgery in RLCs. The guidelines utilized in this study have proven effective. SA can lead to reduced healthcare costs, lower opioid usage, and increased patient turnover. The two cases series demonstrate improved anesthesia outcomes. Future randomized clinical trials with sufficiently large sample sizes are necessary to establish high-quality evidence regarding the safety, efficacy, and cost-effectiveness of SA.

## Introduction

Safe, affordable surgical, and anesthesia care is essential for reducing premature deaths and disabilities, promoting welfare, and driving long-term development. However, resource-limited countries (RLCs) face a large gap in surgical care provision; in Eastern Sub-Saharan Africa, only 20% of surgical needs are met^[1,2]^. This case series has been reported in line with the PROCESS 2023 Guideline and checklist^[3]^.

In Ethiopia, anesthesia services have grown due to new training programs, and neurosurgery has advanced significantly with the establishment of the first neurosurgery residency program in 2006, supported by Ethiopian and Norwegian collaboration^[4,5]^.

Our institution routinely performs various neurosurgical procedures, such as craniotomies, laminectomies, discectomies, and ventriculoperitoneal shunts, primarily under general anesthesia (GA). Although awake craniotomies and lumbar spine surgeries (LSS) are common worldwide, they are less prevalent here, with Ethiopian data indicating that LSS constitutes 21.2% of neurosurgical procedures. The leading indications for neurosurgery in Ethiopia are head injuries, spinal conditions, and tumors^[6,7]^.

Evidence-based practice supports the use of spinal anesthesia (SA) for LSS, despite its limitations. However, our specialized referral teaching hospital has not implemented this practice, potentially due to a lack of clear protocols and prevailing attitudes. In contrast, high-income countries have established protocols for SA in LSS^[5]^.

LSS can be performed under GA or SA, each with distinct benefits and risks. GA is often preferred due to its higher patient acceptance, unlimited anesthesia time and secure airway management, particularly in prone positioning. Nonetheless, current guidelines advocate for SA as a viable option^[8,9]^.

For RLCs, prioritizing investments in cost-effective surgical care is essential. Performing LSS under SA has proven successful in various healthcare settings, significantly reducing perioperative costs by minimizing anesthetic drug use and potentially eliminating the need for post-anesthesia care unit (PACU) stays^[10-12]^.

Research indicates that SA may offer advantages over GA, such as shorter surgery and anesthesia durations without compromising hemodynamic stability, thus enhancing overall efficiency in the operating room (OR)^[13]^.

Many studies discuss the benefits of SA in LSS. Key findings indicate that SA is associated with reduced postoperative pain and a lower requirement for pain medications in PACU. Patients also experience fewer instances of nausea and vomiting, leading to higher overall satisfaction. Importantly, SA does not increase the risk of complications such as urinary retention, bradycardia, or hypotension^[7]^.

For patients with significant cardiovascular risks, SA is deemed safe and maintains stable hemodynamics throughout the procedure. Compared to GA, SA requires fewer medications and less frequent use of vasoactive agents, enhancing safety by minimizing medication errors and reducing the risk of pathogen transmission^[10,12,14]^.

Despite the routine use of SA, the authors note that no other cases of LSS under SA have been reported at their institution. A recent study in Ethiopia highlighted the competency of anesthesia students in administering SA, achieving a success rate above 80%^[15]^. The manuscript emphasizes the importance of implementing guidelines and protocols to promote the use of SA in LICs, where it has proven effective.

## Statues of anesthesia in relation to spinal surgery

The manuscript examines the growing preference for SA over GA for shorter LSS lasting less than 3–4 hours. SA offers advantages such as avoiding intubation and mechanical ventilation, thus reducing the risk of cardiopulmonary complications. Some medical centers are utilizing targeted nerve blocks, such as erector spinae and thoracolumbar interfacial plane blocks, to enhance patient comfort and recovery, although their routine application is limited by equipment availability^[16,17]^.

Additionally, the case series highlights the cost disparity between GA and SA, noting that GA can be more than twice as expensive in both public and private hospitals. SA is commonly used in various spinal procedures, including awake surgeries like laminectomy, discectomy, fusion, and lumbar fusion. At the authors’ center, LSS, both open and endoscopic, are frequently performed under GA for conditions such as disc degeneration, herniation, and stenosis^[6,7,18]^.

## Protocol

### Patient selection

While there are no specific studies identifying the ideal candidate for SA, certain relative contraindications exist for its use in spine surgery. The indications and contraindications for awake spine surgery are summarized in Table 1. Ultimately, the decision to use local or regional anesthesia should be guided by the clinical judgment and comfort level of both the surgical and anesthesia teams.

**Table 1:** Indication and contraindication for awake spinal surgery with permission taken from Fiani *et al*^[18]^.

Indication	Contraindication
Surgeries involving a maximum of two vertebrae levels	Surgeries involving > 2 vertebrae
Surgery that are minimally invasive or utilize endoscopic technique	Surgeries with unpredictable durations
Surgeries requiring neural feedback	Patient with risks of respiratory compromise
Aging populations	High BMI
Patients deterred from general anesthesia	Obstructive sleep apnea
	Preexisting anxiety or depression
	Bleeding disorder or coagulopathies
	Intracranial hypertension
	Failed back syndrome
	Radiological demonstration of arachnoiditis or severe spinal stenosis
	Smoking

During the pre-anesthesia evaluation, a detailed discussion with the patient is essential to plan the use of SA for LSS. Full patient consent, including written informed consent, is required.

### Anesthesia protocol

#### Preoperative

Preoperative analgesia delivery is a component of pre-emptive analgesia. For preemptive anesthesia, we advise dexamethasone 8 mg intravenously (IV), non-steroid anti-inflammatory drugs (NSAIDs) such Diclofenac, meloxicam, and acetaminophen 1000 mg IV three times a day (TID) including the postoperative phase. Typically, 5–10 mg of IV diazepam is administered 30 minutes before SA for anxiolytic effects. The surgical team uses lidocaine 2% with adrenaline to skin infiltrates the lumbar operative site.

### Preoperative indication and contraindication

#### Intraoperative

At our institute, we typically use 0.5% heavy bupivacaine combined with fentanyl. Once in the OR, patients receive a SA at the L3-4 or L4-5 (lumbar spine) level, using 12.5–15 mg of heavy bupivacaine with 10 µg of fentanyl. This provides a dense block of the lower thoracic and entire lumbar region, lasting for more than 4 hours. Our sedation protocol includes the use of benzodiazepines, with 5 mg of diazepam, despite the drawback of delayed recovery since Midazolam, lorazepam, and propofol infusions are not always available. At the end of the procedure, the surgical team infiltrates the bilateral deep lumbar facet joints and the cutaneous tissue with 0.25% isobaric bupivacaine for post-operative pain.

#### Postoperative

**Figure 1.**
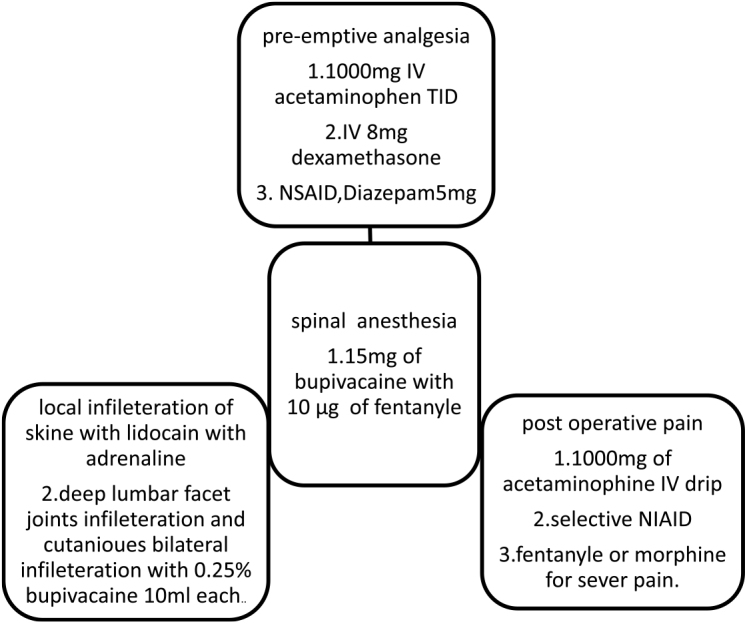
The summary of the anesthesia protocol for LSS performed by SA.

The combined effect of SA, infiltration of the skin and surrounding facet joints, along with IV infusion of acetaminophen and the NSAID/diclofenac, typically facilitates a smooth recovery. Acetaminophen and NSAIDs are administered routinely as the foundation of pain management, with opioids like fentanyl or morphine reserved for cases of severe pain. The protocol is summarized (Fig. 1)

### Positioning protocol

The SA is usually administered at a table near the main operating table. Once the SA is injected, the patient is immediately positioned supine with the head elevated. The patient remains in this position for 10–15 minutes while a urinary catheter is inserted. After the SA has taken effect, the patient is repositioned prone in preparation for surgery.

A soft pillow is used to relieve pressure on the abdomen, ensuring that the testes, breasts, and eyes are protected. The arms rest on soft arm supports with less than 90 degrees of flexion. The patient’s head is adequately supported with a cotton pillow for comfort. The surgeon then infiltrates the surgical site with 2% lidocaine.

The patient is allowed to settle into a comfortable position before the surgical site is prepped. Active communication with the patient may be maintained throughout this process.

### Airway management

The patient’s airway is handled while they are in the prone position, and because they are only slightly sedated, they should continue to breathe on their own. A nasal catheter is offered for the administration of oxygen. A complete tracheal intubation set with laryngeal mask airway will be ready in advance for any emergency airway management.

### Postoperative course

#### Pain control

The nurse or the neurosurgery resident should follow the pharmacological postoperative anesthesia regimen and should also implement non pharmaceutical measures to improve comfort. Heat and Cold alternative therapy may be used no more than 20 min per hour for up to 4 consecutive hours. This helps reduce muscle discomfort and speed up recovery^[19]^. The study use numerical pain rate score (NPRS) pain assessment tools which is reliable and appropriate in clinical practice .The NPRS is a 0 to 10 point scale where 0 indicate no pain and 10 score is the extremes pain as bad as it could be, or worst pain^[20]^.

## Illustrative case series

The following case series demonstrate two examples of SA, highlighting the benefits of SA. Case 1 involves a 35-year-femal who underwent L5–S1 Laminectomy and Discectomy for Herniated lumbar disk with Radiculopathy and Case 2 presents a 58-year-old male who underwent a L3-L5 decompression LSS.

## Case 1

### Preoperative course

**Figure 2.**
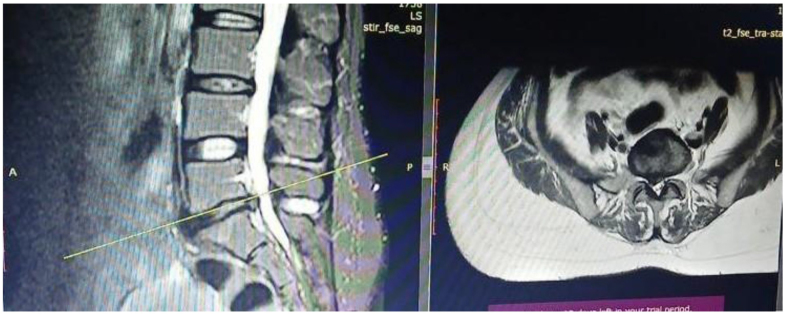
CT scan of case 1 with herniation of lumbar disk with radiculopathy.

A 35-year-old female patient presented with low back pain due to foraminal stenosis, radiating bilaterally to the legs, accompanied by numbness and tingling sensations for the past 2 years. She has no history of trauma or chronic medical conditions. The patient reports progressive weakness in the lower extremities, with worsening pain while walking. Physical therapy, injections, and NSAID have not provided significant relief. A computerized tomography (CT) scan revealed an L5-S1 herniated lumbar disc with radiculopathy and focal protrusion of the disc margin lateral to the intervertebral foramen (Fig. 2).

### Operative details

Since the surgery involved only two vertebral levels, SA was chosen during the pre-anesthesia period after discussion with the patient. Prior to the procedure, the patient was administered 1000 mg of acetaminophen via IV drip, 8 mg of dexamethasone, and 5 mg of diazepam. In the OR, while the patient was in a sitting position, 15 mg of heavy bupivacaine with 10 µg of fentanyl was administered as a spinal block at the L3-L4 level by senior anesthetist. Ten minutes later both sensory and motor blocks were achieved below the T4 (thoracic spine) level. The patient was positioned prone on the operating table, supported with pillow pads at the nipples and anterior superior iliac crest, allowing the abdomen to remain free. The arms were positioned in 90-degree abduction and flexion.

**Figure 3.**
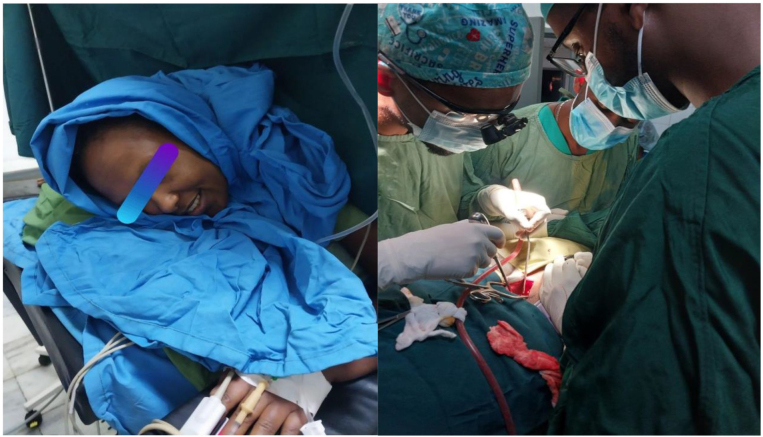
Patient comfortably positioned while surgery undergo.

The surgery lasted 3 hours and 5 minutes, with an estimated blood loss of less than 300 mL. The patient remained comfortable throughout the procedure (Fig. 3). The anesthesia team routinely used “verbocaine” a local term referring to reducing the patient’s stress, anxiety, and pain through conversation. There were no significant changes in the patient’s systolic, diastolic blood pressure, mean arterial pressure (MAP), pulse rate, and oxygen saturation, all records of vital sign was within normal range (Table 2).

**Table 2:**
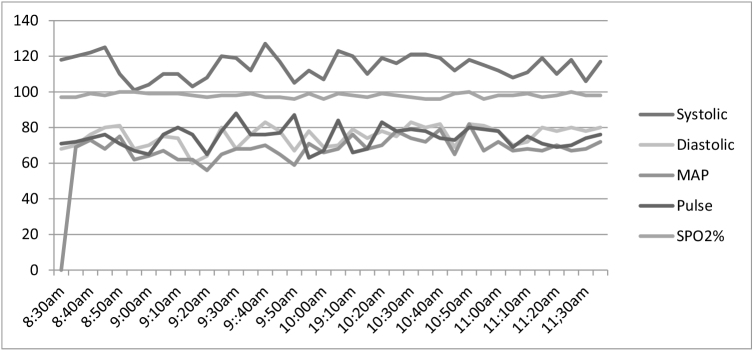
Three hours intra-operative systolic, diastolic, MAP, pulse rate, and oxygen saturation record of patient, taken from patient monitor.

### Postoperative period

The wound was then cleaned, local infiltration to central and medial part of facet joints. With bupivacaine 0.25% of 20 mL done, wound was closed and covered followed by skin infiltration with the same agent. There were no intraoperative anesthesia and surgery complications during the procedure, the patient stay In PACU, for 2 hour before transfer to regular ward. While in PACU the patient had good pain control requiring only one dose of intramuscular dicofenac 75 mg.

## Case 2

### Preoperative course

A 58-year-old man presented with a three-year history of lower extremity pain, tingling, and cramping sensations. His symptoms worsen with standing or walking but are typically relieved by leaning forward or sitting. Conservative treatments have not provided adequate relief, and he has been on Nifedipine for hypertension for the past 2 years. The patient has a family history of complications related to GA.

Magnetic resonance imaging revealed multi-level spinal stenosis in the lumbar region, particularly at the L3-L4 and L4-L5 levels. X-rays showed L3 on L4 and multi-level degenerative disc disease throughout the cervical, thoracic, and lumbar spine. Based on these findings, the surgical team recommended a decompressed laminectomy. After discussing the options, the patient expressed a preference for SA for the surgery.

### Operative details

The patient received an IV drip of 1000 mg of acetaminophen, 5 mg of diazepam, and 8 mg of dexamethasone as priming dose in the OR. A SA in sitting position at the L2–3 level with 0.5% bupivacaine and 10 µg of fentanyl produced sensory loss and a motor block below the T4 level.

The patient was placed prone on the operating table, with the abdomen left free, with pillow pads supporting the nipples and anterior superior iliac crest spine. The arms are flexed and abducted to a 90° angle. Before the procedure begins, the incision site skin infiltrated with 2% lidocaine and adrenaline. Classical open decompressive laminectomy performed. After that, prior to the wound closing, local infiltrations were performed using 0.25% bupivacaine of 20 mL to the medial and central facet joints the wound was cleansed, sutured, and covered followed by cutaneous infiltration . There was very little blood loss and no anesthetic or surgical intraoperative complication. Surgery took only 2 hour. There was no pain or discomfort reported by patient intraoperative. Blood pressure, pulse, oxygen saturation, temperature, and electrocardiograph were with in normal value intra operatively and in PACU.

### Post-operative course

The patient vital sign and pain monitored before being moved to the regular ward, no derangement in vital sign, pain assessed every 30 minutes for 3.5 hours in PACU, there was only mild discomfort which was relieved with NSAID after 3.5 hour (Table 3).

**Table 3.**
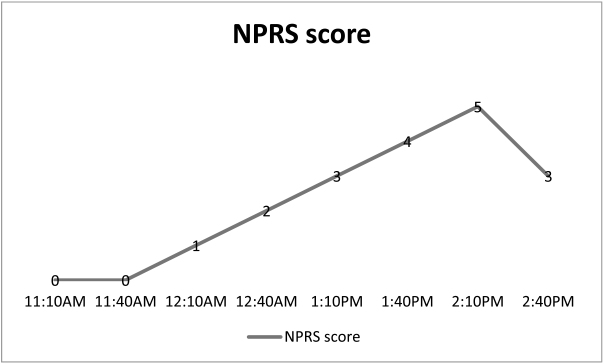
Pain assessment score using NPRS every 30 minutes for 4 hours in the PACU.

## Discussion

The use of SA for LSS is challenging but offers numerous benefits for both patients and healthcare systems, especially in RLCs. SA provides significant advantages, particularly by avoiding the risks and negative outcomes associated with GA. There is growing recognition of the side effects of GA, such as opioid use and cardiopulmonary complications, which are often more severe in elderly patients^[18,21]^. In contrast, SA significantly reduces these risks, making it an excellent option for older patients undergoing LSS.

In our case series, neither patient experienced complications or side effects, and both had effective pain control without the need for opioids. Additionally, SA has been linked to shorter hospital stays, lower healthcare costs, and a reduced incidence of complications such as surgical site infections^[21]^, The study noticed the anesthesia cost of SA were lower compare to GA making it especially beneficial in RLCs likes ours.

According to the American Society of Anesthesiologists (ASA), patients with a physical status of ASA ≥ 3 are at higher risk of postoperative complications during spinal procedures under GA. However, Khan *et al* reported that patients with multiple comorbidities (ASA ≥ 3) are low-risk candidates for SA, with outcomes comparable to those of patients with ASA < 3 undergoing GA^[22]^.

In our study, the second patient was considered high risk for GA due to multiple comorbidities, including cardiovascular disease and immediate family history of anesthetic complications under GA. The use of SA, combined with pre- and postoperative infiltration of the skin and surrounding facet joints, proved particularly beneficial. This protocol provides optimal preoperative, intraoperative, and postoperative care for patients under SA for LSS.

Although some anesthetists may prefer GA over SA due to concerns about unplanned surgery prolongation or patient discomfort, SA is becoming the standard practice in developed countries^[23]^. RLCs could greatly benefit from this approach, as it reduces healthcare costs, optimizes hospital bed utilization, and leverages easily available resources^[10,15]^.

## Conclusion

Our initial experience showed that starting a SA program for LSS is feasible. It may reduce healthcare costs, increase patient satisfaction, and improve patient pain control. Additionally, patients with comorbidities who are not ideal candidates for GA could benefit from this approach. SA also serves as a valuable tool in combating the opioid epidemic. However, randomized clinical trials with sufficiently large sample sizes are needed to provide robust evidence regarding the safety, efficacy, and cost-effectiveness of SA.

## Data Availability

Available upon reasonable request.

## Research registration unique identifying number (UIN)

Unique identifying number is: researchregistry10733, and the registration link is: https://www.researchregistry.com/browse-the-registry#home
